# Extracorporeal membrane oxygenation therapy for severe influenza-associated pulmonary aspergillosis: a case report

**DOI:** 10.3389/fmed.2026.1732770

**Published:** 2026-02-24

**Authors:** Cuijie Tian, Chaofan Liang, Xinyan Zhang, Xiaoju Zhang

**Affiliations:** 1 Department of Respiratory and Critical Care Medicine, Henan Provincial People’s Hospital, Zhengzhou, Henan, China; 2 Department of Respiratory and Critical Care Medicine, Zhengzhou University People’s Hospital, Henan Provincial People’s Hospital, Zhengzhou, Henan, China

**Keywords:** bronchoscopy, critical care, extracorporeal membrane oxygenation, influenza, influenza-associated pulmonary aspergillosis

## Abstract

**Background:**

Influenza-associated pulmonary aspergillosis (IAPA) is an increasingly recognized life-threatening condition in non-classically immunocompromised hosts. This report describes the clinical course and management of severe IAPA involving significant airway invasion.

**Case description:**

A 34-year-old female presented with a 1-week history of fever and rapidly progressive dyspnea. Laboratory testing confirmed influenza A (H1N1) infection and significantly elevated serum galactomannan (GM) index 5.436. Despite invasive mechanical ventilation, the patient developed refractory acute respiratory failure (ARF) characterized by extreme airway resistance (*190 cmH_2_O/L/s*) and extensive subcutaneous emphysema. Veno-venous extracorporeal membrane oxygenation (VV-ECMO) was initiated as a bridge to facilitate diagnostic bronchoscopy, which revealed extensive necrotic pseudomembrane. Subsequent BALF microscopy and mNGS confirmed *Aspergillus fumigatus* coinfection with Influenza A (H1N1), establishing the diagnosis of IAPA. Management involved a combination of systemic and localized bronchoscopic antifungal therapy (Amphotericin B) and iterative airway clearance. Despite complications including secondary extensively drug-resistant Pseudomonas aeruginosa infection and suspected drug-induced hemolysis, the patient was successfully weaned from ECMO on 22 March 2023 and eventually discharged following clinical improvement.

**Conclusion:**

VV-ECMO combined with repeated bronchoscopic interventions can serve as a rescue strategy in selected patients with airway-invasive IAPA and refractory respiratory failure.

## Introduction

Influenza-associated pulmonary aspergillosis (IAPA) has emerged as a severe complication in critically ill patients with influenza, and it may occur even in the absence of classical immunocompromising conditions. Seasonal influenza causes an estimated 1 billion infections annually, with 3–5 million severe cases and 290,000–650,000 respiratory deaths worldwide (1).

Observational cohort studies indicate that the incidence of IAPA among patients with influenza admitted to the intensive care unit (ICU) is substantial and that outcomes are poor. The reported incidence varies widely, ranging from approximately 14% in non-immunocompromised patients to up to 32% in immunocompromised ICU populations in a large multicenter cohort, and 90-day mortality is approximately 50% among affected patients (2). Across published ICU series and reviews, fatality often exceeds 50% despite antifungal therapy (3, 4). These data underscore the clinical importance of early recognition and timely intervention.

Importantly, IAPA is not a uniform entity; it encompasses different patterns of disease. In addition to parenchymal involvement, airway-invasive disease (Aspergillus tracheobronchitis, ATB) represents a distinct phenotype characterized by infection confined to, or predominantly involving, the tracheobronchial tree. Typical bronchoscopic appearances include pseudomembrane, ulcerations, plaques, or obstructive lesions (5, 6). Consistent with current expert definitions and ICU-focused consensus, bronchoscopy plays a central role in identifying airway-invasive forms and obtaining lower respiratory tract specimens for mycological testing in suspected IAPA (3, 7).

The pathobiology of influenza provides a plausible substrate for secondary fungal invasion. Influenza-related epithelial injury, impaired mucociliary clearance, and dysregulated immune responses can predispose to invasive Aspergillus infection, as supported by mechanistic reviews and experimental models (4, 8). Clinically, IAPA may progress rapidly, and diagnosis is frequently established within the first days after ICU admission or influenza confirmation in reported cohorts (3, 4). Delayed recognition may result in swift deterioration and poor outcomes, particularly when infection extends beyond the airways into the lung parenchyma.

Given these challenges, establishing a clear diagnostic label and an explicit evidence chain is essential in case-based reports. In our patient, influenza A (H1N1) infection was confirmed by molecular testing of respiratory specimens, and *Aspergillus fumigatus* was identified early from lower respiratory tract samples using direct microscopy and metagenomic next-generation sequencing (mNGS), supported by elevated serum galactomannan. Our diagnostic framing is aligned with recognized consensus definitions for IAPA and contemporary invasive fungal disease definitions (3, 7).

While IAPA cases are documented, we describe a distinct fulminant phenotype characterized by extreme airway resistance. We highlight the use of early veno-venous extracorporeal membrane oxygenation (VV-ECMO) as a crucial bridge to therapy. This strategy facilitated repeated therapeutic bronchoscopy, enabling aggressive airway clearance alongside antifungal treatment. This approach is consistent with ICU management algorithms that consider VV-ECMO as a rescue strategy in refractory respiratory failure in the context of viral pneumonia–associated aspergillosis management pathways (9).

## Clinical data

The patient is a 34-year-old female who presented with a 1-week history of fever, peaking at 39.4 °C, along with chills, frequent coughing, and significant yellow, sticky sputum occasionally streaked with blood. She also reported generalized body aches, fatigue, and two episodes of non-bloody vomiting. Initially, there was no chest pain, dyspnea, abdominal pain, or diarrhea. She had received intravenous treatment at a local clinic for 3 days, but without improvement.

On 5 March 2023, her dyspnea worsened, prompting evaluation at a local hospital, where her peripheral oxygen saturation (SpO_2_) on room air was 85%. Chest CT showed bilateral lung inflammation, and a diagnosis of “lung infection” was made. Treatment with piperacillin-tazobactam was started for infection control, along with expectorants, spasmolytics, and bronchodilators.

On 7 March 2023, serum (1,3)-β-D-glucan was 237.68 pg/mL and galactomannan index was 0.12 (cut-off ≥ 0.5 or ≥ 1.0 depending on assay). Fluconazole was added to the treatment regimen for antifungal therapy. However, the patient continued to experience dyspnea. A follow-up chest CT on 7 March showed extensive subcutaneous emphysema in the neck, anterior chest wall, and mediastinum, as well as two areas of lung consolidation. She was then transferred to our hospital’s emergency department for further evaluation and management.

Upon admission, her temperature was 36.3 °C, pulse 150 bpm, respiratory rate 32 breaths per minute, and blood pressure 159/89 mmHg. She was alert but displayed signs of agitation. Palpable crepitus was noted in the neck and bilateral axillary regions. The patient exhibited dyspnea and orthopnea. Lung auscultation revealed absent breath sounds in both lungs. The heart rate was 150 bpm, regular, with no murmurs detected.

On admission, laboratory tests showed a white blood cell count of 39.28 × 10^9^/L, with a neutrophil ratio of 90% and a lymphocyte count of 0.68 × 10^9^/L. Hemoglobin was 133 g/L, and platelet count was 442 × 10^9^/L. C-reactive protein (CRP) was elevated at 70 mg/L. The β-D-glucan level was 185.53 pg/mL (reference range: <60 pg/mL, negative; 60–100 pg/mL, indeterminate/observation; >100 pg/mL, positive), and the serum GM test was markedly elevated at 5.436 (cut-off ≥ 1.0). Procalcitonin (PCT) was 0.49 ng/mL, and interleukin-6 (IL-6) was 23.95 pg/mL. Liver and kidney function were within normal limits.

**FIGURE 1:**
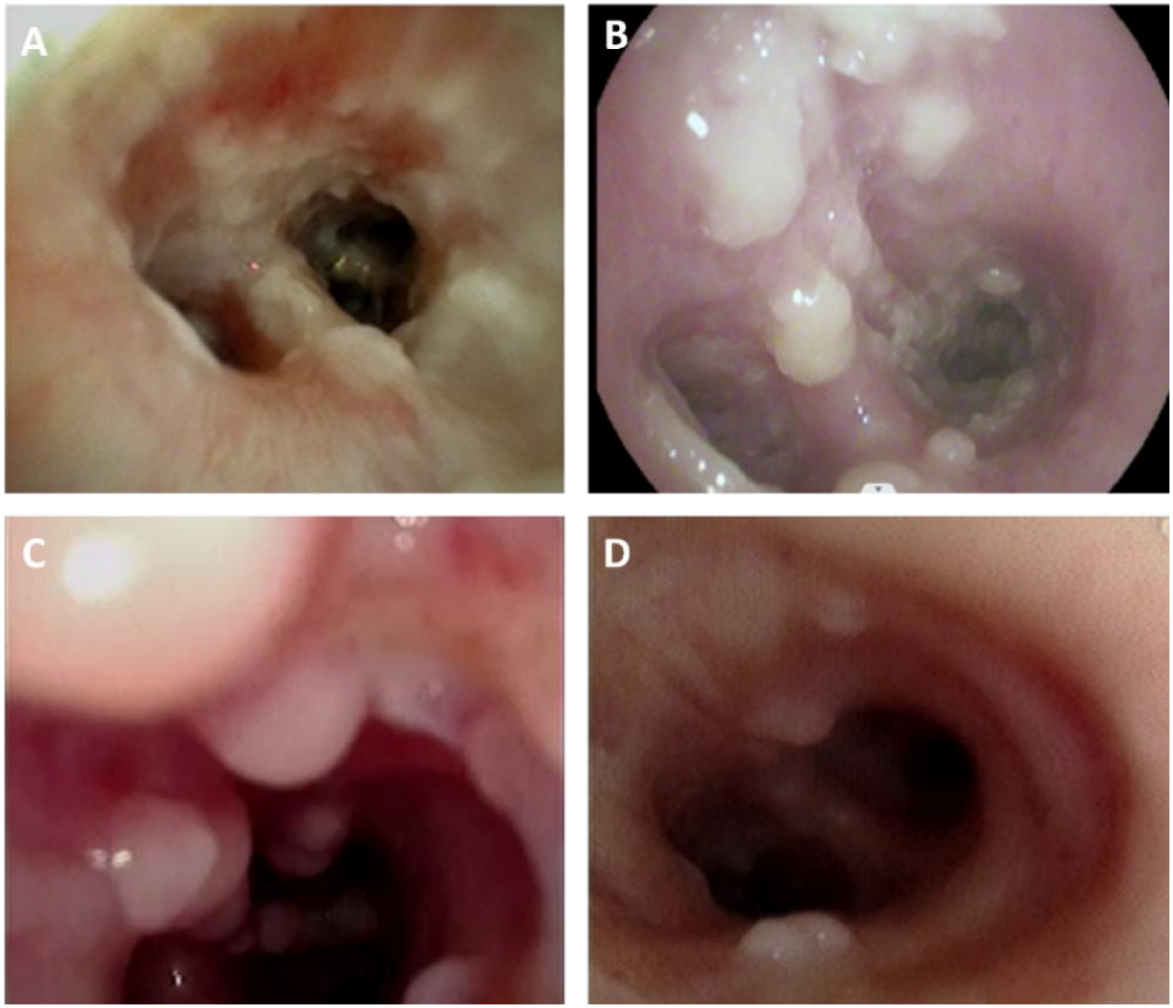
Bronchoscopic appearance of influenza-associated pulmonary aspergillosis (IAPA). **(A)** Bedside bronchoscopy reveals extensive white fungal plaques, pseudomembrane formation, and necrotic material at the opening of the left lower lobe bronchus, with visible mucosal erosion on 08 March 2023. **(B)** Following systemic and initial localized antifungal treatment, multiple polypoid protrusions are observed at the opening of the left lower lobe bronchus on 11 March 2023. **(C)** During treatment, multiple large polypoid nodular protrusions are visible on the mucosa of the right bronchus intermedius, with abundant fungal plaques also present on 11 April 2023. **(D)** After comprehensive systemic and localized antifungal therapy, together with localized steroid treatment, the mucosa of the right bronchus intermedius appears smoother, with a marked reduction in polypoid protrusions on 02 May 2023.

Blood gas analysis upon admission revealed type II respiratory failure. Invasive mechanical ventilation was initiated via orotracheal intubation, with respiratory airway resistance measured at 190 cmH_2_O/L/s and static lung compliance at 14 mL/cmH_2_O. VV-ECMO was employed as life support. Systemic anti-infective therapy included imipenem, linezolid, oseltamivir (for 7 days), voriconazole, and caspofungin. Additionally, localized therapy was administered via nebulization and bronchoscopic amphotericin B instillation. Bronchoscopy revealed extensive white necrotic pseudomembrane (Figures 1A, B). On 9 March, bronchoalveolar lavage fluid (BALF) smear microscopy identified *Aspergillus*, and mNGS confirmed *A. fumigatus* and influenza A virus H1N1. BALF galactomannan index was 4.169 (cut-off ≥ 1.0) and multiple sputum and bronchoalveolar lavage fluid cultures confirmed the presence of *Aspergillus fumigatus*. After IAPA was diagnosed, linezolid was discontinued because there was no microbiological evidence of ongoing Gram-positive infection, and antibacterial therapy was subsequently de-escalated. Serial sputum and BALF cultures were obtained for surveillance, while systemic antifungal therapy with adjunct local (nebulized) antifungal treatment was continued.

**FIGURE 2:**
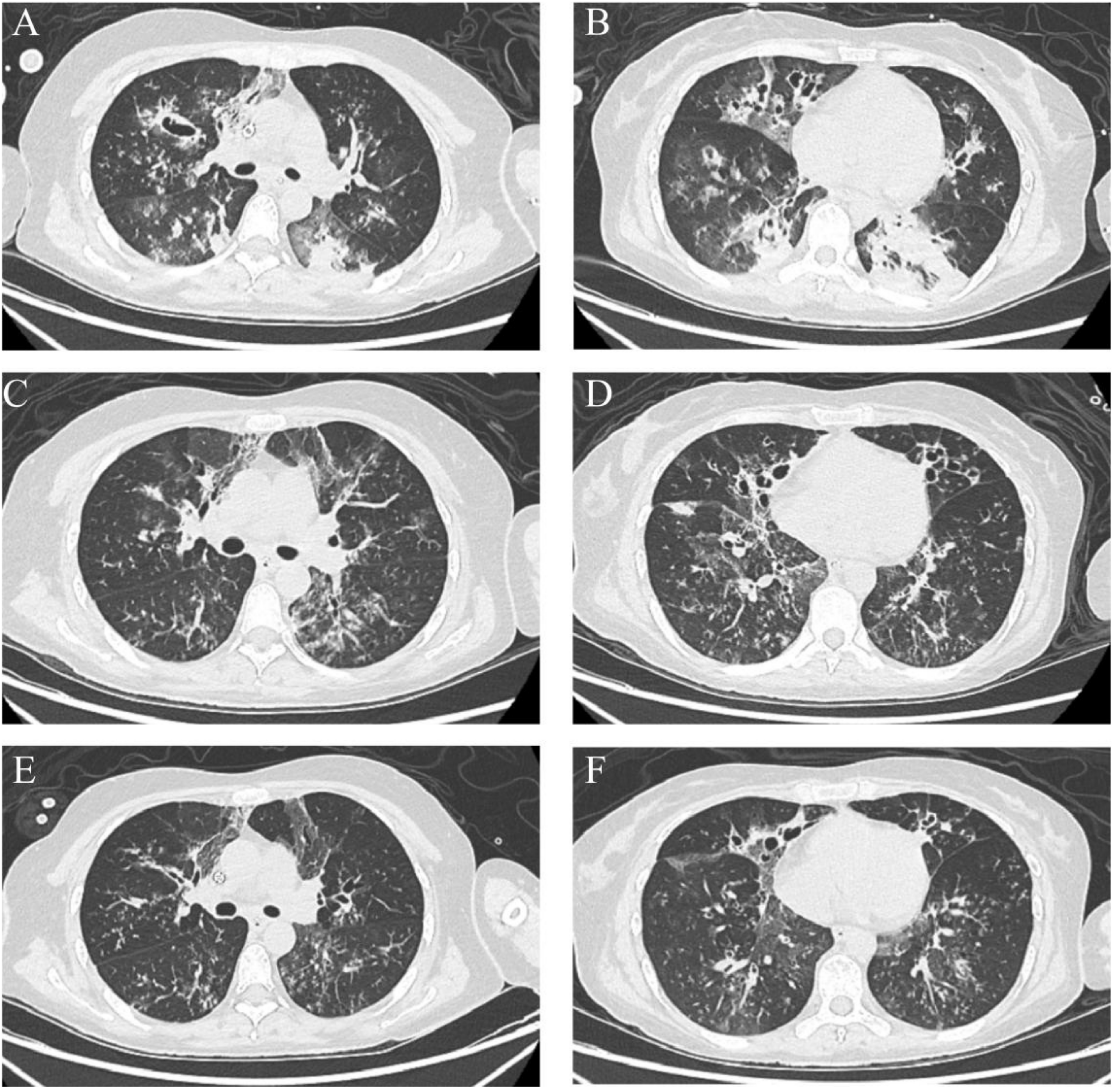
Chest CT showing interval changes in bilateral bronchiectasis. **(A,B)** Multifocal bilateral cylindrical/cystic bronchiectasis with bronchial wall thickening and surrounding patchy opacities on 20 March 2023. **(C,D)** Persistent multifocal bronchiectasis with wall thickening and patchy peribronchial opacities, showing interval improvement compared with the prior scan on 12 April 2023. **(E,F)** Further interval improvement of the peribronchial patchy opacities, with residual bronchiectasis and bronchial wall thickening on 28 April 2023.

Upon admission, evaluate gastrointestinal, liver, kidney function, and hemodynamics, and initiate enteral nutrition within 24 h of admission. At the same time, tracheotomy was performed to strengthen airway management and reduce airway resistance. Continuous renal replacement therapy (CRRT) was instituted to optimize fluid balance and to support clearance of inflammatory mediators. During VV-ECMO support, the patient developed progressive hyperbilirubinemia (peak total bilirubin 174.8 μmol/L; direct bilirubin 134.4 μmol/L) accompanied by cytopenias, including thrombocytopenia (platelet nadir 65 × 10^9^/L) and anemia (hemoglobin nadir 73 g/L). These abnormalities were considered multifactorial, likely involving infection-related sepsis, physiological stress, and ECMO-associated mechanical hemolysis (indicated by elevated indirect bilirubin and anemia). Given the predominance of direct hyperbilirubinemia and the risk of drug-induced liver injury (DILI). Therapeutic drug monitoring showed a supratherapeutic voriconazole concentration of 6.6 μg/mL. Therefore, voriconazole was switched to posaconazole to reduce hepatic burden. After replacement of the membrane oxygenator and supportive management, liver function indices, hemoglobin, and platelet counts improved. A chest CT scan was obtained on 20 March 2023 (Figures 2A, B). Later that night, the patient developed intermittent hypotension consistent with septic shock, clinically attributed to a secondary bacterial superinfection. BALF culture confirmed extensively drug-resistant (XDR) *Pseudomonas aeruginosa*, susceptible only to ceftazidime-avibactam and polymyxin. Guided by susceptibility testing, imipenem–cilastatin was discontinued, and targeted salvage therapy with ceftazidime-avibactam combined with polymyxin B was initiated. Following effective antimicrobial coverage and fluid resuscitation, hemodynamic stability was rapidly restored. Given the concomitant improvement in lung compliance and gas exchange, the patient was successfully weaned off ECMO on 22 March. By discharge, over 20 bronchoscopy procedures had been performed. This aggressive interventional strategy was critical for source control, primarily by mechanically clearing necrotic fungal debris to relieve airway obstruction and facilitating the delivery of localized antifungal medications.

After ECMO removal, high-dose sedative and analgesic medications were used to manage the patient’s dyspnea. A lumbar puncture was performed to rule out central nervous system issues, revealing intracranial pressure of 190 mmH_2_O. Routine cerebrospinal fluid analysis and mNGS results were normal. An electroencephalogram (EEG) indicated background waves consistent with NREM stages II and III sleep. These findings suggested that the altered mental status was likely drug-related. With gradual reduction and discontinuation of medications, the patient regained consciousness, and limb muscle strength normalized.

**FIGURE 3:**
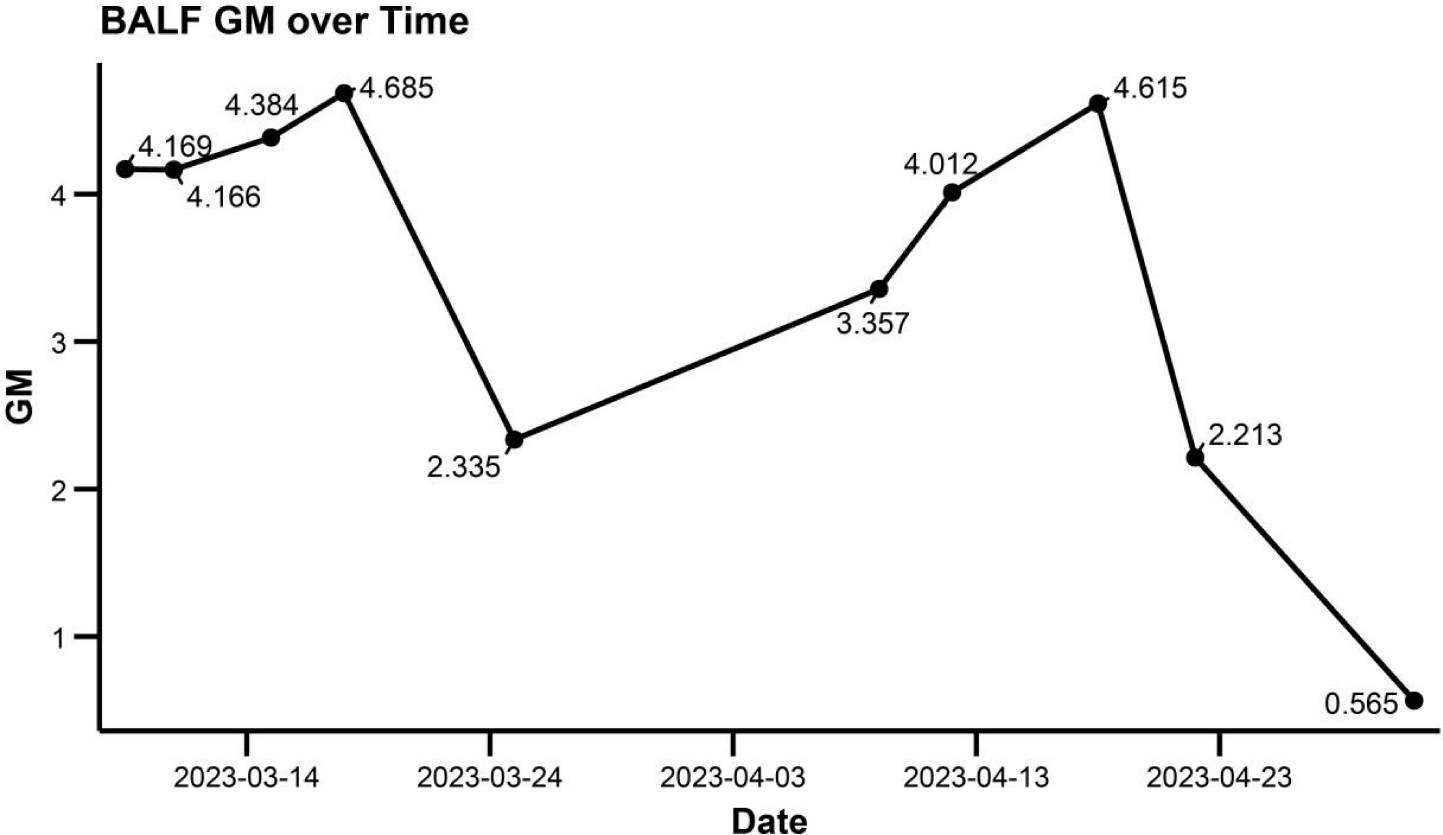
Dynamic changes in bronchoalveolar lavage fluid (BALF) galactomannan (GM) are shown across the treatment course and were used to support antifungal escalation and therapeutic monitoring.

Antibacterial agents were discontinued after repeated sputum and BALF cultures became negative. Two weeks later (11 April 2023), bronchoscopy revealed multiple polypoid mucosal nodules (Figure 1C). Respiratory mechanics showed increased airway resistance (36 cmH_2_O/L/s) and a static lung compliance of 40 mL/cmH_2_O. Repeat inflammatory markers showed no substantial change from prior values, and sputum cultures remained negative. Follow-up bronchoscopy revealed that the multiple polypoid mucosal nodules had alleviated (Figure 1D), while the patchy consolidation on chest CT improved (Figures 2C, D). A subsequent chest CT on April 28, 2023 showed further interval improvement of the peribronchial patchy opacities, with residual bronchiectasis and bronchial wall thickening (Figures 2E, F). These findings collectively suggested that an active bacterial infection was unlikely at that time. The posaconazole trough concentration was subtherapeutic (0.9 μg/mL; target > 1 μg/mL), and BALF galactomannan demonstrated an upward trend (Figure 3). Considering the poor effectiveness of antifungal treatment, posaconazole was switched to voriconazole, and the dose was titrated to achieve serum concentrations of 4.13–5.29 μg/mL (Figure 3). Intravenous methylprednisolone (20 mg daily) was administered. Caspofungin was discontinued once therapeutic azole exposure was achieved.

The patient subsequently developed gastrointestinal bleeding, accompanied by a renewed increase in inflammatory markers. On 17 April 2023, BALF culture grew extensively drug-resistant Pseudomonas aeruginosa; treatment with ceftazidime-avibactam plus polymyxin B was initiated, and nebulized polymyxin E (colistimethate sodium) was administered concurrently. Systemic corticosteroids were discontinued, and high-dose nebulized budesonide (4 mg every 8 h) and continuous nebulized amphotericin B were started. After multiple consecutive negative sputum cultures, ceftazidime-avibactam and polymyxin B were stopped, and nebulized polymyxin E was continued.

**FIGURE 4:**
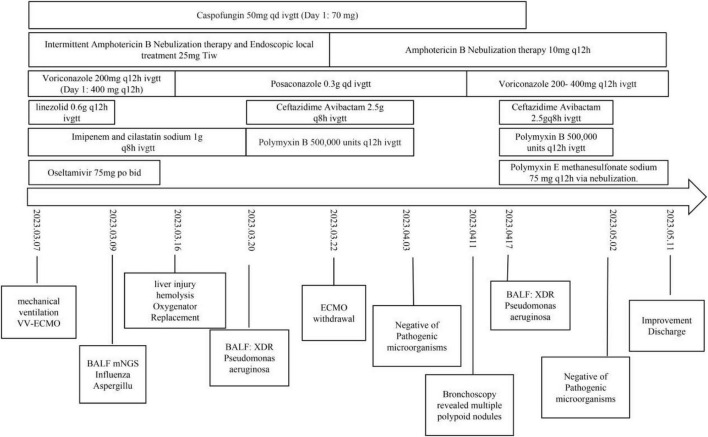
Clinical timeline of key interventions, antimicrobial therapy, microbiological results, and major events during the intensive care unit (ICU) course.

Intermittent renal replacement therapy (IRRT) was administered and ventilatory support was gradually reduced with rehabilitation-focused weaning. On 11 May 2023, the patient was successfully liberated from mechanical ventilation. Subsequently, she was transferred to a local hospital for continued rehabilitation and antifungal therapy. Key clinical events and antimicrobial regimens are summarized in Figure 4. During the follow-up period at the local hospital, voriconazole maintenance therapy was continued for 3 months.

## Discussion

Severe IAPA in non-immunocompromised hosts is an under-recognized and life-threatening clinical entity. It is distinct from the invasive pulmonary aspergillosis (IPA) typically seen in classical immunocompromised populations, such as those with leukemia, HIV/AIDS, or recipients of hematopoietic stem cell or solid organ transplants. Emerging evidence suggests that influenza-induced immunological disruption can impair mucosal immune function, thereby increasing susceptibility to fungal invasion.

In this case, severe airway obstruction was the predominant clinical manifestation, reflecting the unique pathophysiological profile of airway-invasive IPA. In this phenotype, extensive fungal proliferation and invasion of the bronchial mucosal epithelium, triggering severe local inflammation, excessive mucus production, and bronchial spasms. This progression often leads to acute respiratory failure (ARF) that is refractory to conventional mechanical ventilation due to prohibitively high airway pressures and ventilation-perfusion mismatch. Such clinical phenotypes challenge traditional paradigms of IPA and underscore the necessity for heightened clinical vigilance in non-immunocompromised patients presenting with unexplained, severe ARF.

Veno-venous extracorporeal membrane oxygenation may be considered as rescue support in selected patients with severe, potentially reversible acute respiratory failure that remains refractory to optimal conventional management. In our case, VV-ECMO provided physiologic stabilization and helped mitigate ventilator-induced lung injury thereby ensuring the safety of early and repeated bronchoscopic evaluation and airway-directed interventions, which were crucial for source control, diagnosis, and ongoing management of airway-invasive disease. Because this report describes a single case, the potential benefit of VV-ECMO and the overall management strategy cannot be generalized. Clinical decisions should therefore be individualized on the basis of the patient’s condition, institutional expertise, and resource availability.

This report has several limitations. First, this study describes a single patient, and causal inferences regarding the effectiveness of VV-ECMO, repeated bronchoscopy, or specific antifungal regimens cannot be established. Second, histopathological confirmation was not available. However, the diagnosis was robustly supported by combined clinical, bronchoscopic, and microbiological evidence. Third, mNGS results may vary across platforms and lack standardized diagnostic thresholds, limiting generalizability. Finally, the multidisciplinary treatment approach described here may not be universally applicable and should not be extrapolated as a universal management strategy.

## Conclusion

In summary, this case highlights a fulminant, airway-invasive phenotype of IAPA characterized by extreme airway resistance and refractory acute respiratory failure. Early bronchoscopy with comprehensive mycological assessment (including culture/microscopy, mNGS, and galactomannan) was essential for timely diagnosis and for guiding iterative airway-directed interventions. In selected patients with rapidly progressive respiratory failure, VV-ECMO may serve as a rescue bridge that stabilizes gas exchange and facilitates repeated therapeutic bronchoscopy together with combined systemic and localized antifungal therapy. Given the single-case nature of this report, these observations should be interpreted cautiously, and management decisions should be individualized based on patient condition, institutional expertise, and resource availability.

## Data Availability

The original contributions presented in this study are included in this article/supplementary material, further inquiries can be directed to the corresponding author.
